# Genome-scale analysis of genetic regulatory elements in *Streptomyces avermitilis* MA-4680 using transcript boundary information

**DOI:** 10.1186/s12864-022-08314-0

**Published:** 2022-01-21

**Authors:** Yongjae Lee, Namil Lee, Soonkyu Hwang, Woori Kim, Suhyung Cho, Bernhard O. Palsson, Byung-Kwan Cho

**Affiliations:** 1 Department of Biological Sciences, Korea Advanced Institute of Science and Technology, Daejeon, 34141 Republic of Korea; 2 KAIST Institute for the BioCentury, Korea Advanced Institute of Science and Technology, Daejeon, 34141 Republic of Korea; 3 Department of Bioengineering, University of California San Diego, La Jolla, CA 92093 USA; 4 Department of Pediatrics, University of California San Diego, La Jolla, CA 92093 USA; 5 Novo Nordisk Foundation Center for Biosustainability, 2800 Kongens Lyngby, Denmark

**Keywords:** *Streptomyces avermitilis*, Transcription unit architecture, Regulatory elements

## Abstract

**Background:**

The gram-positive bacterium, *Streptomyces avermitilis*, holds industrial importance as the producer of avermectin, a widely used anthelmintic agent, and a heterologous expression host of secondary metabolite-biosynthetic gene clusters. Despite its industrial importance, *S. avermitilis*’ genome organization and regulation of gene expression remain poorly understood. In this study, four different types of Next-Generation Sequencing techniques, including dRNA-Seq, Term-Seq, RNA-Seq and ribosome profiling, were applied to *S. avermitilis* to determine transcription units of *S. avermitilis* at a genome-wide level and elucidate regulatory elements for transcriptional and translational control of individual transcription units.

**Result:**

By applying dRNA-Seq and Term-Seq to *S. avermitilis* MA-4680, a total of 2361 transcription start sites and 2017 transcript 3′-end positions were identified, respectively, leading to determination of 1601 transcription units encoded in *S. avermitilis*’ genome. Cataloguing the transcription units and integrated analysis of multiple high-throughput data types revealed the presence of diverse regulatory elements for gene expression, such as promoters, 5′-UTRs, terminators, 3′-UTRs and riboswitches. The conserved promoter motifs were identified from 2361 transcription start sites as 5′-TANNNT and 5′-BTGACN for the − 10 and − 35 elements, respectively. The − 35 element and spacer lengths between − 10 and − 35 elements were critical for transcriptional regulation of functionally distinct genes, suggesting the involvement of unique sigma factors. In addition, regulatory sequences recognized by antibiotic regulatory proteins were identified from the transcription start site information. Analysis of the 3′-end of RNA transcript revealed that stem structure formation is a major determinant for transcription termination of most transcription units.

**Conclusions:**

The transcription unit architecture elucidated from the transcripts’ boundary information provides insights for unique genetic regulatory mechanisms of *S. avermitilis*. Our findings will elevate *S. avermitilis*’ potential as a production host for a diverse set of secondary metabolites.

**Supplementary Information:**

The online version contains supplementary material available at 10.1186/s12864-022-08314-0.

## Background

Members of the genus *Streptomyces* have been of great interest over the past decades as dominant natural producers of clinically and industrially useful secondary metabolites, including antibiotics and anti-tumour agents [1, 2]. Among streptomycetes, *Streptomyces avermitilis* holds a prominent position as a producer of an important anthelmintics, avermectin [3, 4]. In addition, *S. avermitilis* can serve as a versatile host for the heterologous production of secondary metabolites from other *Streptomyces* species [5]. Such heterologous expression improves the production yield of useful secondary metabolites [6] and enables the production of novel bioactive derivatives of existing secondary metabolites from reconstructed biosynthetic gene clusters (BGCs) [7] .

Recent progress in Next-Generation Sequencing (NGS) techniques have improved our understanding of the genetic background of *Streptomyces* and revealed the presence of uncharacterized potential for secondary metabolite production [8–10]. For *S. avermitilis*, more than 30 BGCs for the production of secondary metabolites are predicted to reside in its genome, further elevating the clinical and industrial potential [9, 11]. However, despite the numerous discoveries of potentially bioactive compounds in *Streptomyces* genomes, many more remain unknown due to the functionally silent nature of biosynthesis genes for secondary metabolites [12, 13]. The emergence of multi-drug resistant bacteria and low productivity of bioactive secondary metabolites has raised the demand for extensive revision and exploration of *Streptomyces* genomes to meet clinical and industrial needs. As a producer and heterologous expression host of important anthelmintics and other secondary metabolites, *S. avermitilis* is worth to be investigated to increase its potential for secondary metabolite production and activate cryptic BGCs. Although diverse regulatory mechanisms of secondary metabolism have been reported, most of these are confined to characterized compounds with proven value [14–16]. The distinct nature of the genus *Streptomyces*, such as its complex life cycle with accompanying morphological and physiological changes [4], GC-rich genome, and enormous coding potential (more than 7000 genes) [9], suggests the presence of unidentified genetic regulatory elements for expression of genes related to secondary and primary metabolism. To start, the interpretation of diverse regulatory elements governing gene expression is required.

Transcription is the first step of gene expression and diverse regulations take place in transcription [17, 18]. Thus, elucidation of transcription unit architecture is important for understanding genetic regulatory mechanisms. In this study, we provide fundamental information on the genome-wide transcription unit (TU) architecture of *S. avermitilis* determined from transcription start site (TSS) and transcript 3′-end position (TEP) information acquired by differential RNA-Seq (dRNA-Seq) and Term-Seq, respectively. dRNA-Seq reveals the 5′-end positions of transcripts and differentiate the TSSs from processed 5′-ends by identifying the presence of 5′-triphosphate, which is a typical characteristic of bacterial primary transcripts. On the other hand, Term-Seq reveals the 3′-ends of transcripts, including transcription termination sites and processed 3′-ends. Those information-rich data sets, regulatory elements identified from the TU architecture along with gene expression data, will enable expanding our knowledge of comprehensive genetic regulatory features in *S. avermitilis* via integrated data analysis [19–24].

## Results

### Genome-wide identification of transcription start sites

**Fig. 1:**
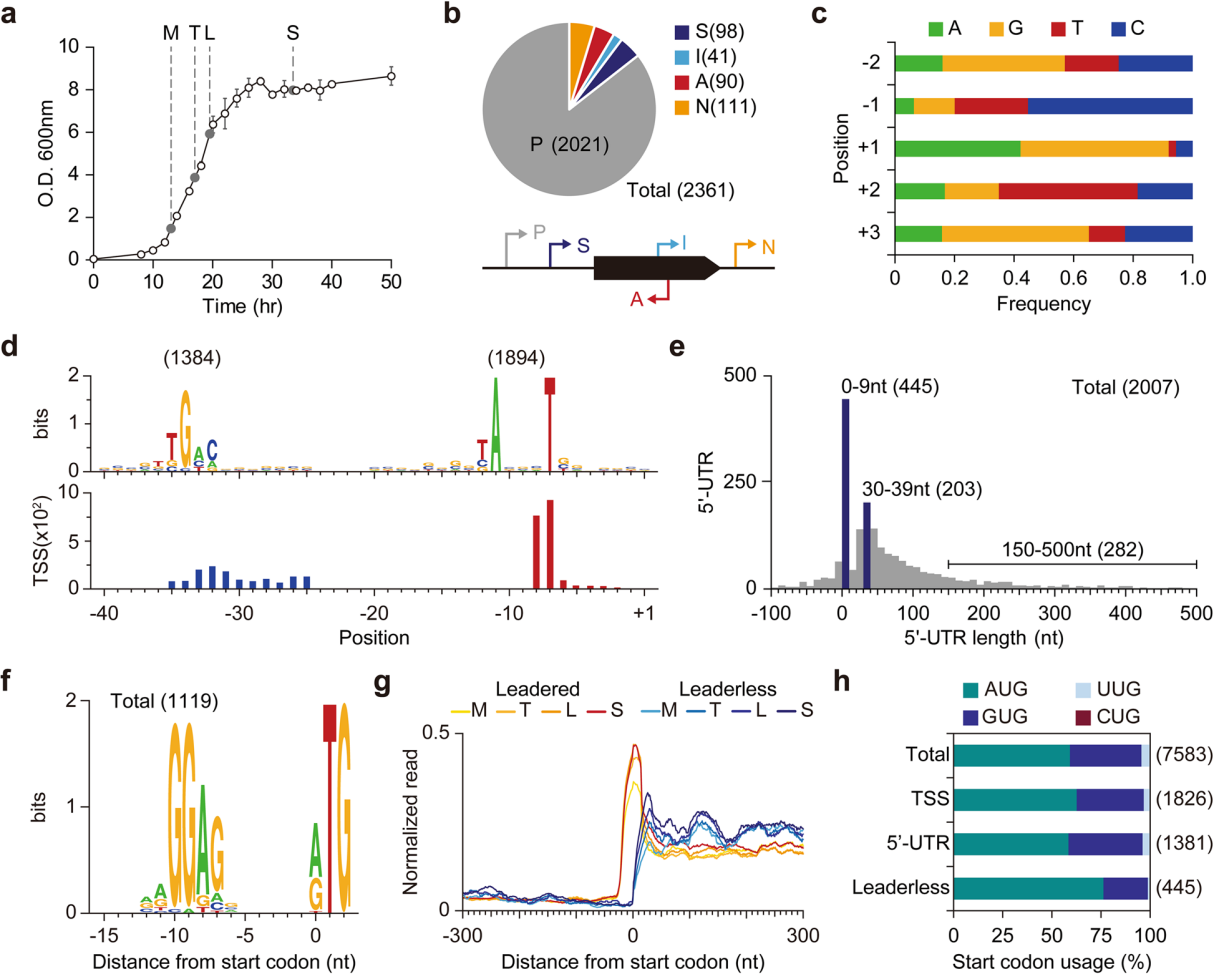
Genome-wide identification of transcription start sites. **a** Growth profile of *S. avermitilis* and sampling time points. Cells were harvested 13, 17, 19.5, and 33.5 h after inoculation for mid exponential, transition, late exponential, and stationary phases, respectively. **b** The number of transcription start sites (TSS) identified in this study. The identified TSSs were classified as either primary (P), secondary (S), internal (I), antisense (A), or intergenic (N) based on the relative position from adjacent genes. **c** Nucleotide frequency near TSSs. **d** Conserved promoter sequence of *S. avermitilis*. Each promoter motif was found separately using MEME suite. **e** The distribution of the 5′-UTR length. The 5′-UTR length was calculated as the distance from primary TSS to its associated CDS. **f** Conserved RBS sequence of *S. avermitilis*. RBS sequence was found with the whole sequences of 5′-UTRs longer than 10 nt. **g** Ribo-Seq read density near start codons of leaderless mRNA and leadered mRNA (**h**) Start codon usage of leaderless mRNA and other mRNA

By exploiting dRNA-Seq, we experimentally identified TSSs in the *S. avermitilis* genome. Briefly, the dRNA-Seq method distinguishes the presence of triphosphate at 5′-ends of bacterial intact primary transcripts from processed or degraded transcripts [25]. Since *Streptomyces* undergo major morphological and physiological changes during growth, samples were prepared from four different growth phases to determine TSSs [26] (Fig. 1a). From the dRNA-Seq, a total of 5.7–11.7 million reads from each sequencing sample were mapped to the genome, with high reproducibility for the two biological replicates (*R* ^2^ > 0.999 for both two sets of libraries). As a result, a total of 2361 TSSs were detected. To validate the detected TSSs, we measured transcriptome under different growth phases of *S. avermitilis* using RNA-Seq and changes of RNA-Seq profile across the determined TSSs were examined. From the RNA-Seq, a total of 12.2–16.8 million reads from each sequencing sample were mapped to the genome with at least 136-fold genome-wide coverage and high strand-specificity (Additional file 1: Fig. S1a). To examine whether the transcriptome varies along the growth phases and the data sets are reproducible, hierarchical clustering and principal component analysis were performed (Additional file 1: Fig. S1b). A significant difference in gene expression between the growth phases was observed and biological replicates conformed to each other. To examine the increment of RNA-Seq profile across the determined TSSs, RNA-Seq read density was calculated for each growth phase. RNA-Seq read density drastically increased across the TSSs for all the four growth phases, indicating that the determined TSSs are bona fide (Additional file 1: Fig. S1c).

Then, the TSSs were classified into five categories based on their relative positions to adjacent genes, which represent the leading genes of candidate TUs (Fig. 1b). Briefly, TSSs located within 500 nt upstream and 100 nt downstream from the start codons of annotated open reading frames (ORFs) were classified as either primary (P) or secondary (S) based on the levels of corresponding read counts. TSSs located within an ORF or in a reverse strand of the annotated ORF were classified as internal (I) or antisense (A), respectively. TSSs that were not classified into these four categories were classified as intergenic (N). Using these criteria, 2021, 98, 41, 90, and 111 TSSs were classified as primary, secondary, internal, antisense, and intergenic TSSs, respectively (Additional file 2: Table S1). The presence of secondary and internal TSSs implies that diverse regulatory modes are present for activation of a gene, and in addition, the presence of antisense and intergenic TSSs may indicate the presence of novel transcripts in the *S. avermitilis* genome.

### Determination of *cis*-regulatory elements

Around the TSSs, diverse sequence elements, including promoters, 5′-untranslated regions (5′-UTRs) and ribosome binding sites (RBSs), are present and those elements direct expression of a gene through transcription and translation. To understand genetic regulation of *S. avermitilis*, identification of those elements is crucial. Near the TSS positions, preference toward specific nucleotides was clearly observed (Fig. 1c). For example, purines were strongly preferred (more than 90%) for the + 1 position. In contrast, pyrimidines were enriched at − 1 and + 2 positions from TSS, and T was a dominant nucleotide for + 2 position despite the high occurrence of G and C in the genome (GC contents = 70.7%) [9] (Fig. 1c). The sequence composition stabilizes the incorporation of + 1 purine nucleotide by base stacking interactions with the adjacent purine bases on the template strand [27]. From the upstream regions of the detected TSSs, we found the highly conserved 5′-TANNNT (*P*-value < 0.05; MEME) and the less-conserved 5′-BTGACN (*P*-value < 0.05; MEME) as the − 10 and − 35 promoter elements, respectively (Fig. 1d). The depicted sequence elements, including promoter structure and nucleotide usage near TSS, were comparable to those reported in other *Streptomyces* species including *S. clavuligerus*, *S. coelicolor* and *S. lividans*, suggesting that fundamental elements required for transcription initiation are highly conserved across the *Streptomyces* genus [26, 28, 29]. The promoter motifs are also comparable to *E. coli*, whose − 10 and − 35 promoter elements are 5′-TATAAT and 5′-TTGACA, respectively, suggesting that *Streptomyces* promoters may function in *E. coli* [30, 31].

The identification of TSS leads to the determination of 5′-UTRs, which typically encode the Shine-Dalgarno sequence for ribosome binding and additional regulatory sequences for modulating translational efficiency [32] and post-transcriptional regulation [33]. From the primary TSSs assigned to coding sequence (CDS), we observed that 5′-UTR lengths are most frequently in a size range of 30–39 nt (Fig. 1e). For mRNAs with 5′-UTR, purine-rich ribosome-binding sequences were found upstream of the start codon (Fig. 1f). Interestingly, the 5′-UTR length distribution showed that a considerable number of leaderless mRNAs (22.2%), whose 5′-UTR length is shorter than 9 nt, are present in the *S. avermitilis* transcriptome (Additional file 2: Table S1). To test whether the leaderless genes are bona fide leaderless or mis-annotated, we additionally measured ribosome-protected mRNA fragments (RPFs) at a genome-wide scale using ribosome profiling [22]. To capture RPFs, the mycelia at different growth phases were treated with the inhibitor of translation elongation (thiostrepton). After disrupting cells by grinding rapidly frozen mycelia in liquid nitrogen, the monosome fraction was isolated from the size exclusion chromatography. After high-throughput sequencing of ribosome-protected mRNA, the reads were mapped to the genome with high reproducibility for the four growth phases (*R* ^2^ = 0.9961, 0.8845, 0.9971 and 0.9997 for mid exponential phase, transition phase, late exponential phase and stationary phase, respectively), and read density across the start codons was calculated for each growth phase. For leaderless genes, the read density drastically increased right after their start codons, whereas sequencing read spanned 5′-UTRs for leadered genes (Fig. 1g). These results indicate that the leaderless genes are truly devoid of 5′-UTRs. Since a leaderless gene is also absent of a RBS, AUG was highly preferred as a start codon compared to mRNA with 5′-UTR, for direct interaction with the anticodon of initiator tRNA [34] (Fig. 1h). Long leader sequences (length of 5′-UTR longer than 150 nt) were found in 282 transcripts (14.0%), suggesting the presence of potential regulatory RNA structures mediating post-transcriptional regulation. Overall, the genomic architecture of *cis*-regulatory elements will serve as a fundamental resource to understand transcriptional and post-transcriptional regulation of *S. avermitilis*.

### Elucidation of diverse *cis*-regulatory sequences for transcription initiation

**Fig. 2:**
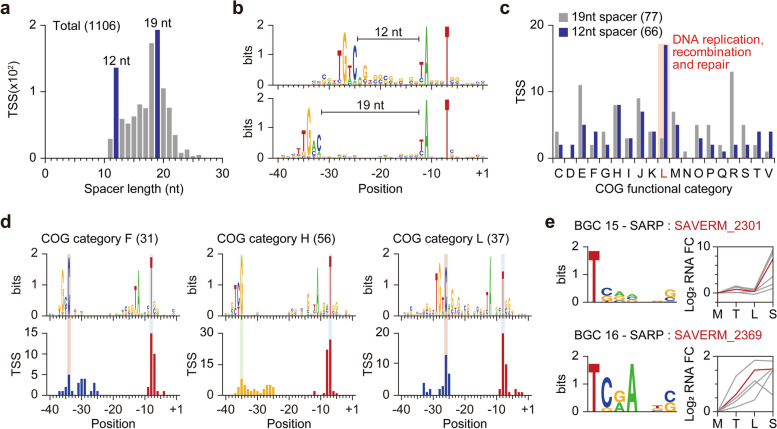
Regulatory elements of transcription initiation. **a** The distribution of spacer length. The spacer length was calculated using the − 35 and − 10 elements concurrently found TSSs. **b** Differential use of promoter sequence based on the spacer length. Each promoter motif was found separately using MEME suite. **c** Genes with 12 nt spacer have distinct function. The COG category was assigned using WebMGA. **d** Promoter sequence of genes assigned to COG functional categories F, H, and L. **e** Expression change of SARP family regulator and its potential target genes and predicted binding motif. Regulators are colored in red. The heptameric binding sites of SARP family regulators were detected using FIMO and only up-regulated genes at stationary phase (*P*-value < 0.05 in DESeq2) were selected to create sequence logo

The *Streptomyces* genome encodes diverse sigma (σ) factors for transcription initiation [9], which recognize unique promoters by interacting mainly with − 35, − 10 elements, and the spacer sequence. The diversity in the spacer lengths and the large numbers of σ factors encoded in the *S. avermitilis* genome (approximately 60 σ factors) suggest the strong dependence on promoters for regulation of transcription initiation [17, 35]. Interestingly, the spacer length distribution showed two distinct peaks of 12 nt and 19 nt (Fig. 2a). Promoters with a 12 nt spacer had 5′-BTGTCV as the conserved − 35 element, rather than 5′-BTGACN (Fig. 2b). As a sigma factor regulates genes of a specific function by recognizing a distinct promoter motif [36], we analyzed the functional differences between genes that have promoters with either 12 nt or 19 nt spacer lengths using Clusters of Orthologous Groups (COG) assignment [37, 38]. Interestingly, genes with a 12 nt spacer were functionally enriched in replication, recombination, and repair compared to genes with a 19 nt spacer (Fig. 2c). We expanded this approach to analyze the promoter sequence conservation of genes with similar function. While − 10 elements (5′-TANNNT) were highly conserved across most of the groups, − 35 elements varied significantly across the functional groups (Fig. 2d; Additional file 1: Fig. S2). The − 35 elements of genes assigned to COG functional categories F (nucleotide transport and metabolism), H (coenzyme transport and metabolism), and L (replication, recombination and repair) were found as 5′-GC/TC, 5′-TCG, and 5′-BTGTCV, respectively. The sequences as well as the positions of − 35 elements were varied. The − 35 elements of genes assigned to COG functional categories F and H were positioned at 34 and 35 nt from TSSs, respectively. In contrast, the − 35 elements of genes assigned to COG functional category L were positioned at 26 nt from TSSs, which is far closer than the position of generally found − 35 element. Thus, diversity in − 35 element sequences and spacer lengths are major *cis*-regulatory elements for differential transcriptional regulation of genes with distinct functions.

As some sigma factors are auto-regulated by themselves [39], the regulons for each sigma factor can be inferred from the TSS information by comparing the promoter sequences of a sigma factor and other genes. Based on the observation that spacer sequences are variable, from − 40 to − 35 region (6 nt) of total TSSs were locally compared to the same region of each sigma factor’s TSS using FIMO [40] and *P*-value was used as the parameter for similarity (1 nt frame-shift was allowed for each comparison). Then the TSSs were clustered based on local *P*-value using *k*-means clustering method and distinct conserved sequences were found for two sigma factors, SAVERM_741 and SAVERM_3117 (Additional file 1: Fig. S3a, b). Interestingly, the putative − 10 element sequences for SAVERM_741, SAVERM_3117, and their potential regulons were distinct from 5′-TANNNT. Moreover, the potential regulon of SAVERM_3117 includes stress response related genes, such as heat shock protein, tellurium resistance protein, phage shock protein A, GroES, and penicillin-binding protein encoded genes.

### Identification of *cis*-regulatory sequences for *Streptomyces* antibiotic regulatory proteins

*Streptomyces* possess various secondary metabolite gene clusters in the genome with specific regulatory mechanisms for each cluster. To identify potential regulatory features of secondary metabolism, we searched for potential transcription activators present in each secondary metabolite gene cluster using InterPro [41]. Among the regulatory genes identified by AntiSMASH [11], SAVERM_410, SAVERM_2301, SAVERM_2369, and SAVERM_3632, which are predicted to be located in type I polyketide (filipin, 100% similarity), type I polyketide-butyrolactone-other polyketide (chlorizidine A, 11% similarity), type II polyketide-type I polyketide-other polyketide (mannopeptimycin, 14% similarity) and ladderane-arylpolyene-nonribosomal peptide (WS9326, 22% similarity) BGCs, respectively, had bacterial transcriptional activator domains (BTAD). The three regulators of uncharacterized BGCs, SAVERM_2301, SAVERM_2369 and SAVERM_3632, seem to be associated with previously proposed polyketide BGC (SAVERM_2277 ~ 2282), polyketide BGC (SAVERM_2367 ~ 2369) and nonribosomal peptide BGC (SAVERM_3636 ~ 3651), respectively, considering the genomic positions [6, 42], and analysis on the predicted regulators for uncharacterized BGCs could extend our understanding on the secondary metabolism of *S. avermitilis*. Commonly, *OmpR*/*PhoB*-type DNA-binding domains were found near the N-termini of the four predicted regulators. These properties are well-conserved in *Streptomyces* antibiotic regulatory proteins (SARP), whose binding sites typically display heptameric repeats [43]. To identify the binding sites of the SARP-family regulators, we collected the reported binding sites of other SARP-family proteins [43–48] and predicted the potential binding sites of the identified SARPs within each secondary metabolite gene cluster based on the sequence conservation (Additional file 1: Fig. S4a). From the identified TSSs, the 150 nt upstream sequence of each TSS (36 TSSs in total) in each secondary metabolite gene cluster containing a putative SARP family regulator was collected and heptameric binding sequences were predicted using FIMO [40]. Only heptameric repeats with 4 nt or 15 nt spacing, which is the typical spacing length of SARP recognition sequences [43], were considered as potential SARP binding sites. As a result, potential binding sites for three of the four SARP family regulators were identified (Fig. 2e; Additional file 1: Fig. S4b). SAVERM_410 related binding sites were not identified because the genes within the same secondary metabolite gene cluster were transcriptionally silent and, as a result, no TSSs were found within the cluster.

To test whether the identified SARP family regulators activate other genes in the same BGCs, expression changes of the SARP family regulators and their potential regulons were monitored. RNA-Seq data was normalized by DEseq2 [49] to calculate expression fold changes of each gene in transition phase, late exponential phase, and stationary phase compared to mid exponential phase with statistical significance (Additional file 3: Table S2). We compared the expression pattern of the identified SARP family regulators, SAVERM_2301, SAVERM_2369, and SAVERM_3632, with their potential target genes (Fig. 2e; Additional file 1: Fig. S4c). SAVERM_2301 showed great accordance in gene expression pattern with its potential targets. For SAVERM_2369, about half of the potential target genes showed similar gene expression patterns, while only one potential target gene showed a similar gene expression pattern to SAVERM_3632. The poor correlation between expression levels of SAVERM_3632 and its corresponding putative target genes is likely due to the poor expression level of SAVERM_3632 or the presence of the additional domain, nucleotide-binding adaptor shared by APAF-1, certain resistance gene products, and CED-4 (NB-ARC) at the C-terminal region of SAVERM_3632, suggesting the presence of additional regulatory elements [50]. Taken together, this suggests diverse regulatory modules including pathway specific regulators as well as σ factors are involved in differential control of transcription initiation in *S. avermitilis*.

### Genome-wide identification of 3′-end positions of RNA transcripts

**Fig. 3:**
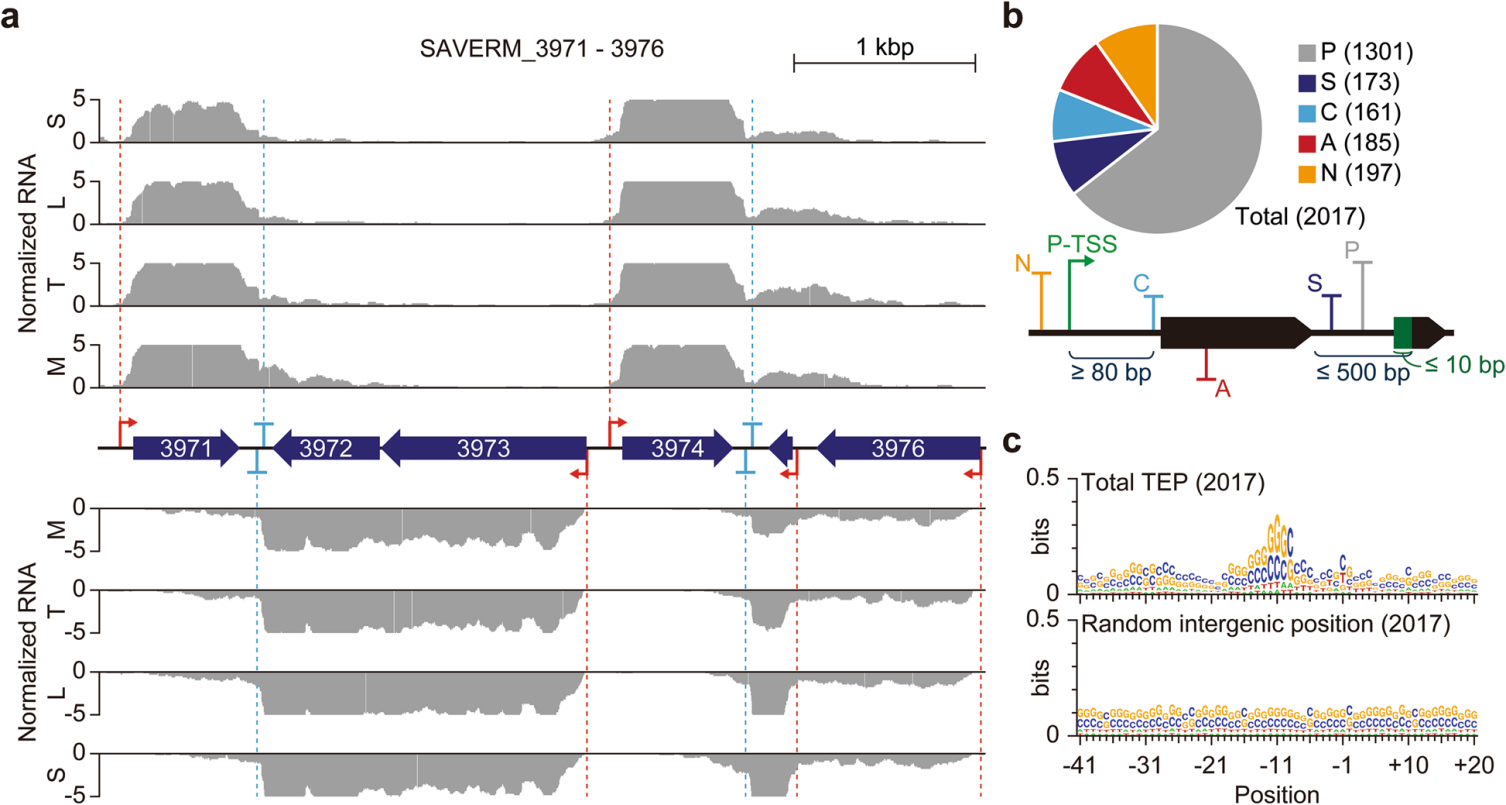
Genome-wide identification of transcript 3′-end positions (TEP). **a** Example of detected TSSs, TEPs, and corresponding RNA-Seq profiles. **b** The number of TEPs identified in this study. The identified TEPs were classified as either primary (P), secondary (S), cis-regulatory (C), antisense (A), or intergenic (N) based on the relative position from adjacent genes and TSS. **c** The alignment of sequences near TEPs. The 41 nt upstream to 20 nt downstream sequences of TEP were used for sequence logo generation using Weblogo

For experimental identification of TU architecture, 3′-end positions of RNA transcripts are required in addition to the TSS. To this end, Term-Seq [20] was carried out with high reproducibility (*R* ^2^ = 0.9993 for the biological replicate) to identify transcript 3′-end positions (TEP). As a result, 2017 TEPs were detected, and decrease of RNA-Seq read density coincided with the TEP positions, supporting the accuracy of our TEP determination (Fig. 3a; Additional file 1: Fig. S5a). Then, the TEPs were classified into five categories similar to the TSS classification (Fig. 3b). TEPs located less than 500 nt downstream from the gene were classified as primary or secondary TEP based on their read counts. In contrast, TEPs positioned more than 500 nt downstream from the gene were classified as intergenic. Antisense TEPs were annotated based on the presence of genes on the complementary strand. If the primary TSS of the downstream gene was located upstream of the TEP, the TEP was classified as *cis*-regulatory (note that the minimum distance from primary TSS to *cis*-regulatory TEP was first set to be 80 nt for the proper formation of terminator structure). If multiple *cis*-regulatory TEPs were present for one gene, only the TEPs with the highest read counts were considered to alleviate the complexity of downstream analysis (note that only 9 TEPs were discarded through this step). Under our classification criteria, 1301, 173, 161, 185, and 197 TEPs were classified as primary (**P**), secondary (**S**), *cis*-regulatory (**C**), antisense (**A**), and intergenic (**N**), respectively (Additional file 4: Table S3).

### Elucidation of transcript 3′-end sequences for transcription termination

**Fig. 4:**
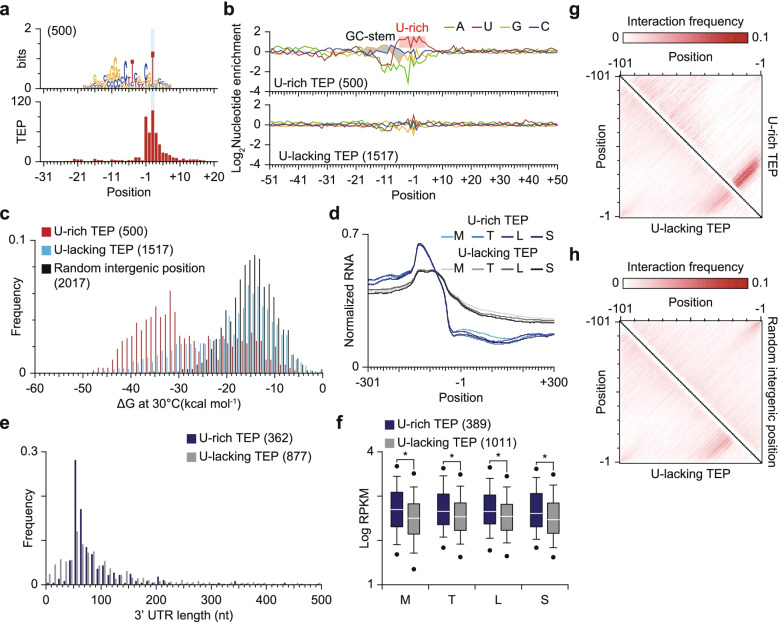
Comparative analysis on the two types of TEPs. **a** Distinct U-rich intrinsic terminator motif. The motif was detected from the 41 nt upstream to 20 nt downstream sequences of TEP using the MEME suite. **b** Nucleotide enrichment analysis of the two types of TEPs. Nucleotide enrichment was calculated by dividing the frequency of each nucleotide of the TEP set with the frequency of the same nucleotide of random intergenic positions. **c** ΔG distribution of TEPs. The ΔG was calculated from 41 nt upstream sequences, including TEPs or randomly selected intergenic positions, using RNAfold with the temperature parameter of 30 °C. **d** RNA-Seq read density near the two types of TEPs. **e** The distribution of the 3′-UTR length. The 3′-UTR length was calculated as the distance from primary TEP to its associated CDS. **f** Differences in expression of genes exploiting two different types of TEPs. RPKM was calculated for each biological replicate and the average value was used. Only 5th and 95th percentile outliers were represented with dots. *P-value < 0.001 (Wilcoxon rank sum test, two-sided) **g** Base interaction frequency comparison of U-rich and U-lacking TEPs. The RNA structure was predicted with 101 nt upstream sequence of each TEP or randomly selected position using RNAfold with the temperature parameter of 30 °C and the interaction frequency of each set of two positions was calculated by dividing the number of observed interactions in the predicted RNA structure with the number of total entries. **h** Base interaction frequency comparison of U-rich and U-lacking TEPs

For bacteria, transcription termination occurs intrinsically without the transcription termination factor, Rho, and GC-rich stem structure followed by a poly U tail is one of the typical sequence elements for those Rho-independent transcription terminations [51, 52]. To investigate whether the Rho-independent transcription termination is prevailed in *S. avermitilis*, nucleotide preference around the TEPs were analyzed (Fig. 3c; Additional file 1: Fig. S5b). The sequence alignment results clearly showed the presence of a palindromic GC-rich sequence, which may form stable stem structure, at the upstream of TEPs (Fig. 3c). However, a poly U tail was not observed across the TEP, indicating that Rho-independent transcription termination is not prevalent in *S. avermitilis* (Additional file 1: Fig. S5b). Since *Streptomyces* possesses a GC-rich genome, stem structure may form at random genomic positions, where there are no transcriptional terminator. To examine any sequence characteristics of transcription terminators, an MEME suite analysis was performed [53] and a U-rich sequence motif was found in about 25% of TEPs (Fig. 4a). Although the level of U occurrence was not high compared to a typical bacterial Rho-independent transcription terminator, U is relatively enriched for those TEPs considering the GC-rich nature of *Streptomyces* genomes [54] (Fig. 4b).

Stable stem structure at the upstream region of TEPs is a key component of Rho-independent transcription termination. To evaluate the potential of stem structure formation at the upstream region of TEPs, ΔG of upstream sequences was calculated. Strikingly, despite the A-U base-pairing interaction being weaker than the G-C base-pairing interaction, ΔG distribution of U-rich TEPs was shifted toward a lower value compared to that of other TEPs lacking U-rich motif (U-lacking TEPs) or random intergenic sequences (Fig. 4c). In contrast, the ΔG distribution of U-lacking TEPs was similar to that of random intergenic sequences. To test whether the U-lacking TEPs are bona fide or not, we sought to analyze the changes in RNA-Seq read density near the TEPs (Fig. 4d). RNA-Seq read density clearly decreased across the both U-rich and U-lacking TEPs, and in addition, the 3′-UTR lengths of the two types of TEPs were similar to each other, indicating that U-lacking TEPs are genuine 3′-ends of transcripts (Fig. 4e). In RNA-Seq read density profiles, the upstream read density value was higher for the U-rich TEPs than for the U-lacking TEPs (Fig. 4d). Moreover, the downstream read density value displayed exactly the opposite trend, suggesting that the U-rich TEPs act as stronger transcription terminators than the U-lacking TEPs and thus, expression of genes utilizing the U-rich TEPs is higher than that of genes with the U-lacking TEPs. To test this hypothesis, the reads per kilobase per million (RPKM) values of genes with different types of TEPs were compared. The RPKM values of genes exploiting the U-rich TEPs were higher than those of genes exploiting the U-lacking TEPs across all four growth phases (Fig. 4f).

Overall, our data strongly support that the detected TEPs are bona fide and TEPs with a U-rich motif determine 3′-boundaries of transcripts more strictly. However, the differences between the U-lacking TEPs and random intergenic positions are not obvious. The GC-rich nature of the genome sequence may result in low ΔG value for random positions, and thus, the ΔG distribution of random positions could be similar to the ΔG distribution of U-lacking TEPs regardless of their actual RNA structure. For a better understanding of the properties of the U-lacking TEPs, we compared the upstream RNA structure of those TEPs to the upstream RNA structure of the randomly selected intergenic sites. We calculated the interaction frequency of each set of two positions deduced from the predicted RNA structure and displayed this in a matrix (Fig. 4g, h). We observed highly conserved interactions that likely induce the formation of stable stem structure for U-rich TEPs. To a lesser extent, similar interactions were present for the U-lacking TEPs (Fig. 4g). However, for the random intergenic sites, this interaction was not observed (Fig. 4h). Presumably, formation of stem structure is a key determinant for determining transcript 3′-boundaries and most U-lacking TEPs are highly likely to be derived from different transcription termination mechanisms, such as Rho-dependent transcription termination [55, 56] or RNA processing [52].

### Determination of transcript boundaries leads to identification of transcription units

**Fig. 5:**
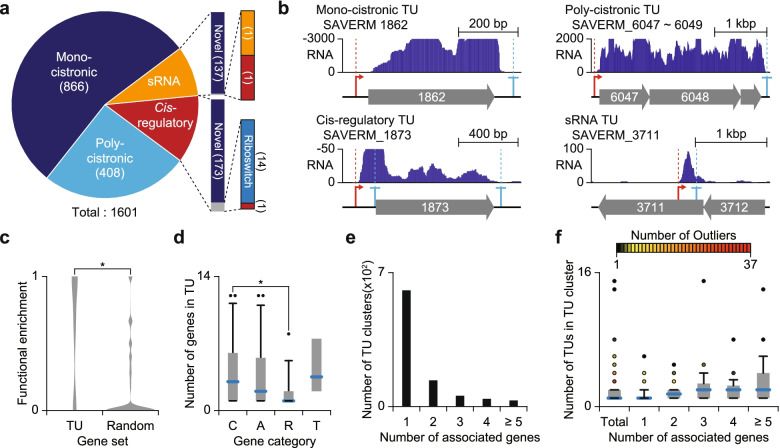
Genome-wide determination of transcription units. **a** The number of determined TUs from TSSs and TEPs. The determined TUs were classified as either mono-cistronic, poly-cistronic, *cis*-regulatory, or small RNA TU based on the number of associated genes or the distance from TSS to downstream gene. The identified *cis*-regulatory TUs or small RNA TUs were searched against the Rfam database. **b** Examples of TU types identified in this study. **c** Functional relatedness of genes belonging to same poly-cistronic TU. The functional enrichment was defined as the maximum frequency of genes with same COG functional category in a single poly-cistronic TU. *P-value < 0.001 (Wilcoxon rank sum test, two-sided) (**d**) Number of genes within a TU containing a certain gene in a secondary metabolite biosynthesis gene cluster. C, A, R, and T represent core, additional, regulatory, and transport related gene containing TU, respectively. *P-value < 0.05 (Wilcoxon rank sum test, two-sided) (**e**) The number of TU clusters containing certain number of genes. **f** The number of TU in a TU cluster

We determined about 2 thousand TSSs and TEPs in the *S. avermitilis* genome, respectively, enabling us to determine a total of 1601 TUs by linking TSSs to TEPs (refer to Methods for detailed explanation) (Additional file 5: Table S4). The determined TUs were classified as mono-cistronic TU, poly-cistronic TU, *cis*-regulatory TU and sRNA TU (Fig. 5a, b). In particular, the identified sRNA and *cis*-regulatory TUs were searched against the Rfam database [57]. Among the identified 188 *cis*-regulatory TUs, 14 TUs were found to be riboswitches and one TU was found to be the *cis*-regulatory element. The detected riboswitches and *cis*-regulatory element include glycine riboswitch ahead of *gcvT* (SAVERM_2773) and actino-pnp *cis*-regulatory element ahead of *pnp* (SAVERM_2523), which are widely conserved among *Streptomyces* [58, 59]. For sRNA TUs, only two of 139 were matched to MS_IGR-5 type sRNA and 6C type *cis*-regulatory element, which are conserved in Actinobacteria [60, 61]. Among the 32 *cis*-regulatory elements including riboswitches deposited in the Rfam database [57], both TSSs and TEPs were found in 28 riboswitches, indicating the powerful detection performance of our experiments.

Determination of TUs revealed that genes are transcribed in the form of either mono-cistronic or poly-cistronic transcripts. While mono-cistronic transcripts may assure individual and subtle modulation of the genes, poly-cistronic TUs may enable rapid and simultaneous regulation on genes for the related cellular functions such as secondary metabolite biosynthesis. We compared the functions of the genes in a single poly-cistronic TU to the functions of randomly selected genes based on COG assignment [37, 38]. Genes belonging to a same poly-cistronic TU were functionally related with each other compared to randomly selected genes (Wilcoxon rank sum test, two-sided *c* 0.001, Fig. 5c). Among the mono-cistronic and poly-cistronic TUs, in particular, 148 TUs were located in secondary metabolite BGCs, furthering our understanding of regulation of secondary metabolism related genes (Additional file 1: Fig. S6). The secondary metabolite products such as polyketide and non-ribosomal peptides are occasionally synthesized with multiple genes. The genes related to biosynthesis are often in proximity with each other on the genome, suggesting that the genes are likely to be transcribed in a single transcript. In contrast, regulation related genes are likely to be transcribed separately to regulate other genes in the biosynthesis gene cluster. The number of genes in a TU containing genes with distinct functions was calculated, and TUs containing core genes were composed of more genes than TUs containing regulatory genes (Wilcoxon rank sum test, two-sided *P* < 0.05, Fig. 5d).

Among the determined mono-cistronic or poly-cistronic TUs, certain sets of TUs shared the same genes with each other, likely resulting from use of alternative TSS or TEP, or post transcriptional processing. The existence of TU variants on a certain gene suggests that complex transcriptional regulation is present to fine-tune the expression of the gene under a certain condition. To elucidate the comprehensive landscape of TU architecture, the maximal set of TUs sharing common genes was defined as a ‘TU cluster’ referring to a previous approach [62]. A total 865 TU clusters were determined, and most of them contained only one gene, indicating that most genes are transcribed independently (Fig. 5e). Transcription of multiple genes in a single transcript may serve as an efficient strategy to regulate expression of multiple genes with a limited number of regulatory modules, however, poly-cistronic TUs may not be favourable for fine-tuning the expression of each gene, requiring additional transcription events or post-transcriptional processing, which may result in subordinate TUs. The number of TUs in a TU cluster was generally proportional to the number of genes in the TU cluster (Fig. 5f). Overall, the high-throughput determination of TUs gives insight into the diverse transcriptional regulatory information including *cis*-regulatory elements and the composition of genes that undergo the same regulation for modulation of gene expression or stoichiometry of functionally related proteins.

## Discussion

The bacterial transcripts contain not only protein-encoded genes but also diverse features modulating the expression of proteins in both transcription and translation levels [32, 33, 63]. To fully understand the diverse and complex regulatory mechanisms for gene expression, careful examination on transcription is required since transcription is the first step for gene expression, and defining the 5′ and 3′ boundaries of transcripts, where major transcriptional regulation takes place in, is a top priority. Precise positions of 5′ and 3′-ends of each transcript offer TU information that leads to the identification of diverse regulatory elements [64] and novel transcripts, as well as the fundamental components of transcription, the promoters and terminators [20, 26, 28]. In this study, we applied dRNA-Seq and Term-Seq to *S. avermitilis* for high-throughput detection of TSSs and TEPs at single base resolution, respectively, followed by the determination of TU architecture with their diverse regulatory elements. From the determined TSSs and TEPs, conserved regulatory elements of transcription initiation and termination for individual TUs were resolved.

The conserved promoter structure of *S. avermitilis* showed great concordance with the promoter structure of *S. coelicolor*, suggesting that fundamentals of transcription are highly conserved across the genus *Streptomyces* [26]. The promoter sequences are also similar to other bacteria such as *E. coli*, however, the − 35 element sequence of *Streptomyces* seems more variable, considering the high enrichment of all six nucleotides, 5′-TTGACA, in the − 35 element of *E. coli* [30]. Considering the similarity of promoter sequences with other bacteria, the variability in the − 35 element sequence of *Streptomyces* would be beneficial for expression of heterologous proteins utilizing the native promoters of other bacteria [31]. For transcription initiation, the − 10 elements of promoters were more conserved than the − 35 elements and the functions of corresponding genes in the TU were highly related with selection of − 35 elements with spacer sequence between the − 10 and − 35 elements. This relationship between gene functions and the − 35 elements and the spacer will serve as an efficient strategy for synchronized regulation of multiple TUs by limited numbers of regulatory proteins such as σ factors, enabling the rapid and economical cellular response to environmental changes. Promoter sequence analysis of genes related to secondary metabolism showed less-conserved − 35 elements than others, suggesting that a specific stimulus is required for activation of each secondary metabolite gene cluster. This possibility is further supported by the diverse σ factors (about 60) encoded in its genome, a number far greater than the average number of σ factors in most bacterial genomes [65]. In addition to the sigma factors, the presence of pathway specific regulators contributes to the complex regulation of secondary metabolism. The expression pattern of SARP family regulators and secondary metabolic genes implies that multiple transcription factors, including sigma factors and pathway specific activators, are required for proper onset of secondary metabolism (Additional file 1: Fig. S4c).

Rho-independent transcription termination is more frequently observed than Rho-dependent transcription termination in bacterial cells, and stable RNA secondary structure followed by a stretch of U or, to a lesser extent, without the U-rich sequence, are observed upstream of intrinsic terminators [66]. Moreover, a recent report on *Escherichia coli* revealed that stable RNA secondary structure is observed even for Rho-dependent transcription termination sites, as a protectant for RNA decay [56]. In that sense, a transcription termination mechanism for *Streptomyces* with abundant G and C residues in the genome (more than 70%) is of great interest. Term-Seq analysis revealed that the U-rich TEPs induced more effective transcription termination than the U-lacking TEPs. Moreover, the U-rich TEPs were preferred for highly expressed genes, which may have resulted from the necessity to prevent incidental activation of downstream genes. On the other hand, the utilization of a stable U-rich intrinsic terminator may result in higher gene expression by preventing RNA decay. Despite the similarity in ΔG values of the U-lacking TEPs and random intergenic positions, however, the decreases in RNA-Seq read count across the TEPs clearly support that these TEPs are bona fide 3′ boundaries of transcripts (Fig. 4d).

## Conclusions

In this study, the TU architecture of *S. avermitilis* was elucidated by defining transcripts’ boundaries using dRNA-Seq and Term-Seq. Diverse sequence elements recognized by transcriptional regulators, including sigma factors and transcription factors, were identified from the TSS information. In addition, TEP information suggests a distinct motif for transcription termination in *Streptomyces*. The TU architecture provides insights for unique genetic regulatory mechanisms, as well as the fundamental procedures of transcription in *Streptomyces*, and the homogeneity of the multi-omics data generated in this single study strongly supports those observations. Moreover, by integrating with transcriptome data of varying growth phases provided in this study, we can identify genetic parts for modulating gene expression, such as promoters and terminators, and such components will expand the potential of *S. avermitilis* as a production host for diverse secondary metabolites.

## Methods

### Strain and culture condition

The mycelium of *S. avermitilis* MA4680 (a kind gift from Prof. Jae Kyung Sohng, Sun Moon University) was maintained in 25% glycerol. Cells were first recovered in 250 mL baffled flask containing 50 mL R5− media and 8 g glass beads (3 ± 0.3 mm diameter) at 30 °C, 250 rpm. R5− medium consisted of 5.73 g/L TES (pH 7.2), 103 g/L sucrose, 10 g/L glucose, 5 g/L yeast extract, 10.12 g/L MgCl_2_∙6H_2_O, 0.25 g/L K_2_SO_4_, 0.1 g/L casamino acids, 0.08 mg/L ZnCl_2_, 0.4 mg/L FeCl_3_∙6H_2_O, 0.02 mg/L CuCl_2_∙2H_2_O, 0.02 mg/L MnCl_2_∙4H_2_O, 0.02 mg/L Na_2_B_4_O_7_∙10H_2_O, and 0.02 mg/L (NH_4_)_6_Mo_7_O_24_∙4H_2_O. The inoculum was then transferred to fresh R5− media with 8 g glass beads for main culture. The optical density at 600 nm was measured in biological triplicate with 2 h intervals for growth profiling (first 8 h was skipped due to lag phase). For RNA-Seq, dRNA-Seq and Term-Seq, cultures were sampled at 13, 17, 19.5 and 33.5 h after inoculation for mid exponential, transition, late exponential, and stationary phases, respectively. For ribosome profiling, culture was treated with thiostrepton for 5 min before harvesting the cells. All the cultures for NGS library construction were prepared as biological duplicate.

### RNA-Seq library preparation

RNA-Seq libraries were prepared as previously described [28]. Harvested cells were washed with polysome buffer (20 mM Tris-HCl pH 7.5, 140 mM NaCl, 5 mM MgCl_2_), and then resuspended with lysis buffer (0.3 M sodium acetate pH 5.2, 10 mM EDTA, 1% Triton X-100). The cell suspension was then frozen with liquid nitrogen, and lysed by grinding using mortar and pestle. The cell lysate was centrifuged at 4 °C for 10 min at 16,000×*g* and the supernatant was stored at − 80 °C until used for RNA extraction. RNA was extracted by mixing with equal volume of phenol:chloroform:isoamyl alcohol = 25:24:1 solution. The mixture was then centrifuged and the upper aqueous phase was recovered. DNase I treatment was used to eliminate DNA contaminant in the sample (New England Biolabs). Ribosomal RNA (rRNA) was depleted with Ribo-Zero rRNA Removal Kit Bacteria (Epicentre) according to the manufacturer’s instructions. The rRNA-depleted RNAs were visualized with 2% agarose gel electrophoresis for quality control. RNA-Seq libraries were constructed using TruSeq Stranded mRNA Library Prep Kit (Illumina).

### dRNA-Seq library preparation

dRNA-Seq libraries were prepared as previously described [28]. About 700 ng rRNA-depleted RNA was incubated in 1× RNA 5′ polyphosphatase (TAP) (Epicentre) reaction buffer and 1 U of SUPERase-In (Invitrogen) at 37 °C for 1 h with [TAP(+)] or without [TAP(−)] 1 U of TAP. After ethanol precipitation, 5 pmol of 5′ RNA adaptor (5′-ACACUCUUUCCCUACACGACGCUCUUCCGAUCU-3′) was ligated to the purified RNA with T4 RNA ligase (Thermo) in 1× RNA ligase buffer and 0.1 mg/mL BSA by incubating at 37 °C for 90 min. The adaptor-ligated RNA was then purified using Agencourt AMPure XP beads (Beckman Coulter) according to the manufacturer’s instructions. The purified product was reverse-transcribed using SuperScript III Reverse Transcriptase (Invitrogen) and purified using Agencourt AMPure XP beads. The purified cDNA was amplified and indexed using Phusion High-Fidelity DNA Polymerase (Thermo) for the Illumina sequencing. The amplification step was monitored using a CFX96 Real-Time PCR Detection System (Bio-Rad) and stopped before the PCR reaction was fully saturated. Finally, the amplified library was purified using Agencourt AMPure XP beads, and the concentration of the library was measured with Qubit 2.0 fluorometer (Invitrogen). The size distribution of the library was checked with gel electrophoresis on 2% agarose gel.

### Ribosome profiling library preparation

Ribosome profiling libraries were prepared as previously described [28]. Thiostrepton (20 μg/mL final concentration) was treated for 5 min prior to harvesting cells to inhibit translation elongation. The cell pellet was washed with polysome buffer (20 mM Tris-HCl pH 7.4, 140 mM NaCl, 5 mM MgCl_2_, and 33.5 μg/mL thiostrepton) and resuspended with lysis buffer (475 μL Polysome buffer, 25 μL Triton X-100, and 6 μL DNase I). The cell suspension was frozen with liquid nitrogen and lysed by grinding using mortar and pestle. The cell lysate was centrifuged at 4 °C for 10 min at 16,000×*g* and soluble supernatant was recovered. Ribosome unprotected RNA was digested by treating RNase I (Invitrogen) by incubating at 37 °C for 45 min. After RNase I digestion, RNase was inactivated by treatment with SUPERase-In and monosomes were recovered using a Sephacryl S-400 column (GE Healthcare Life Science). Ribosome protected RNA was recovered using phenol:chloroform:isoamyl alcohol = 25:24:1 solution and rRNA was removed with Ribo-Zero rRNA Removal Kit Bacteria (Epicentre) according to the manufacturer’s instructions. After rRNA depletion, RNA was resolved on a 15% TBE-urea gel and 26–34 nt RNA fragments were size-selected. The size-selected RNA was eluted in 300 mM sodium acetate pH 5.2, 1 mM EDTA and 0.25% SDS. The eluted RNA was further purified with ethanol precipitation and libraries were constructed with NEB Next small RNA library prep set according to the manufacturer’s instructions. The constructed libraries were amplified and indexed using Phusion High-Fidelity DNA Polymerase for Illumina sequencing. The amplification step was monitored on a CFX96 Real-Time PCR Detection System (Bio-Rad) and stopped before the PCR reaction was fully saturated. The amplified libraries were further size-selected on 2% agarose gel with MinElute Gel Extraction Kit (Qiagen).

### Term-Seq library preparation

Term-Seq libraries were prepared as previously described [28]. Five microgram of DNase I-treated RNA was treated with Ribo-Zero rRNA Removal Kit (Epicentre) prior to adaptor ligation. Then, 500 ~ 900 ng of the rRNA-depleted RNA was mixed with 1 μL of 150 μM amino-blocked DNA adaptor (5′-p-NNAGATCGGAAGAGCGTCGTGT-3′), 2.5 μL of 10× T4 RNA ligase 1 buffer, 2.5 μL of 10 mM ATP, 2 μL of DMSO, 9.5 μL of 50% PEG8000, and 2.5 μL of T4 RNA ligase 1 (New England BioLabs). The mixture was incubated at 23 °C for 2.5 h, purified with Agencourt AMPure XP beads (Beckman Coulter) and eluted with 9 μL DEPC-treated water. Then the RNA-adaptor ligates were fragmented using fragmentation buffer (Ambion) by incubating at 72 °C for 90 s. After fragmentation, the product was purified with Agencourt AMPure XP beads and eluted with 8 μL DEPC-treated water. The fragmented RNA was reverse transcribed using 1 μL of 10 μM reverse transcription primer (5′-TCTACACTCTTTCCCTACACGACGCTCTTC-3′) with SuperScript III Reverse Transcriptase (Invitrogen) according to the manufacturer’s instructions. After reverse transcription, the cDNA was purified with Agencourt AMPure XP beads and eluted with 5 μL DEPC-treated water. The purified cDNA was subjected to another adaptor ligation as above, with increased incubation time (8 h) and different amino-blocked adaptor sequence (5′-p-NNAGATCGGAAGAGCACACGTCTGAACTCCAGTCAC-3′). After adaptor ligation, the product was purified using Agencourt AMPure XP beads and indexed by PCR for 10 cycles with Phusion High-Fidelity DNA Polymerase using forward (5′-AATGATACGGCGACCACCGAGATCTACACTCTTTCCCTACACGACGCTCT-3′) and reverse (5′-CAAGCAGAAGACGGCATACGAGATNNNNNN (6 nt index) GTGACTGGAGTTCAGAC-3′) primers.

### High-throughput sequencing

All libraries were sequenced using Illumina HiSeq 2500 platform with either 1 × 100 bp (RNA-Seq and dRNA-Seq) or 1× 50 bp (Term-Seq and Ribo-Seq) read length. The reads were trimmed and mapped to the *S. avermitilis* genome (Accession number BA000030.4).

### Identification of transcription start sites

Transcription start sites (TSS) were identified as previously described [26, 67]. The 5′ end position of dRNA-Seq reads from TAP(+) library were considered to be potential TSSs. Briefly, the potential TSSs less than 100 bp apart from the ones located at neighbouring positions were clustered together. Then, the potential TSSs adjacent to other potential TSSs in the same cluster were sub-clustered together based on the standard deviation of their genomic positions (< 10). Only potential TSS clusters with more than three read counts were considered and the potential TSSs with maximum read counts within each sub-cluster were selected as TSSs. Then the read counts of selected TSS positions from TAP(+) and TAP(−) libraries were compared and positions with more read counts in TAP(−) library were discarded. Then, the selected TSSs were manually inspected using the corresponding RNA-Seq profile [26, 28].

### Identification of 3′-end positions of RNA transcripts

The transcript 3′-end positions (TEPs) were determined as previously described [28]. The 3′-end positions of Term-Seq reads, located within intergenic regions (10 bp invasion to downstream gene was allowed), were clustered together based on the distance from adjacent positions (< 10 bp). Within each cluster, the read count of each position was assumed to follow normal distribution and read count enriched positions were deduced by calculating the modified z-score as below.

$$\boldsymbol{\mu} \left(\boldsymbol{r}\left(\boldsymbol{x}\right)\right)=\frac{1}{\boldsymbol{N}\left(\boldsymbol{x}\right)-1}\left(-\boldsymbol{r}\left(\boldsymbol{x}\right)+\sum \limits_{\boldsymbol{y}\in \boldsymbol{C}\left(\boldsymbol{x}\right)}\boldsymbol{r}\left(\boldsymbol{y}\right)\right)$$

$$\boldsymbol{\sigma} \left(\boldsymbol{x}\right)=\sqrt{\boldsymbol{\mu} \left(\boldsymbol{r}{\left(\boldsymbol{x}\right)}^2\right)-\boldsymbol{\mu} {\left(\boldsymbol{r}\left(\boldsymbol{x}\right)\right)}^2}$$

$$\boldsymbol{Z}\left(\boldsymbol{x}\right)=\frac{\boldsymbol{r}\left(\boldsymbol{x}\right)-\boldsymbol{\mu} \left(\boldsymbol{r}\left(\boldsymbol{x}\right)\right)}{\boldsymbol{\sigma} \left(\boldsymbol{x}\right)}$$

***Z***(***x***) is the modified z-score at position ***x***, ***r***(***x***) is the read count of evaluated position ***x***. ***μ***(***r***(***x***)) and ***σ***(***x***) are the mean and standard deviation of read counts of other positions in the cluster except the evaluated position, respectively. ***N***(***x***) is the length of the cluster containing position ***x*** and ***C***(***x***) is the set of positions within the cluster containing position ***x***.

The positions with read counts of less than 3 or modified z-scores less than 3 were discarded. All the procedures above were conducted separately for each biological replicate. Among the remaining positions, the reproducible positions with the highest read count within the intersecting region of clusters from two biological replicate were selected as TEPs. For example, if genomic positions from 103 to 125 were clustered together for one replicate and genomic positions from 113 to 142 were clustered together for another replicate, the potential TEPs with highest read count within the genomic positions from 113 to 125 was selected as the TEP.

### Read density calculation

The RNA-Seq or ribosome profiling read density for a set of positions were calculated as follow. First, for each position in the set, normalized read density was calculated for the 601 relative positions ranging from upstream 300 nt to downstream 300 nt. The read count of each relative position was divided by the highest read count of the 601 relative positions, generating normalized read density ranging from 0 to 1 for each relative position. Then, the normalized read density was averaged for the set of positions.

$$\boldsymbol{d}\left(\boldsymbol{x},\boldsymbol{p}\right)=\frac{\boldsymbol{r}\left(\boldsymbol{p}+\boldsymbol{x}\right)}{\underset{-300\le \boldsymbol{y}\le 300}{\max}\boldsymbol{r}\left(\boldsymbol{p}+\boldsymbol{y}\right)}$$

$$\boldsymbol{D}\left(\boldsymbol{x}\right)=\frac{\sum_{\boldsymbol{p}\in \boldsymbol{P}}\boldsymbol{d}\left(\boldsymbol{x},\boldsymbol{p}\right)}{\boldsymbol{N}\left(\boldsymbol{P}\right)}$$

***d***(***x***, ***p***) is the normalized read density of a relative position ***x*** from the position ***p***, where −300 ≤ ***x*** ≤ 300. ***r***(***p + x***) is the read count of position ***p + x***, and ***D***(***x***) is the final read density of the relative position ***x***. ***P*** is the set of positions and ***N***(***P***) is the number of positions in the set ***P***.

### Motif discovery

MEME suite was utilized for identification of sequence elements [53]. For detection of promoter motifs, sequences from − 20 to + 1 position of each TSS were utilized to identify − 10 elements, and the sequences from − 40 to − 25 position of each TSS were utilized to identify − 35 elements. The two sequence elements were combined and visualized using Weblogo [68]. For terminator sequence analysis, sequences from 41 bp upstream to 20 bp downstream of each TEP were used for sequence alignment and motif discovery, and upstream 41 bp sequences were used for ΔG prediction. The visualization of sequence and prediction of ΔG were performed by using Weblogo [68] and RNAfold [69], respectively.

### Detection of transcription units

Transcription units (TUs) were determined as previously described [28]. Briefly, adjacent TSSs and TEPs were paired together for determination of the TUs. In case of *cis*-regulatory TEPs, they were allowed to form TU only with TSSs assigned to the same gene. To capture the poly-cistronic TUs, the maximum intergenic distance between two adjacent genes was assumed as 500 bp. For primary, secondary and internal TSSs, any combination of TSSs and TEPs was allowed to form TU on condition every intergenic distance in the TU did not exceed 500 bp. For antisense and intergenic TSS, 1 kbp downstream region was scanned for the presence of the TEP or start codon of a gene. TU was then determined if a TEP was present in that region. If the start codon of a gene appeared in that region, TUs were determined by the same method as the primary, secondary or internal TSSs. The determined TUs were then compared to the RNA-Seq profile, informing the removal of false-positives. Any potential TUs supported by TSS, TEP and RNA-Seq profile not detected from computational processes were manually inspected. The determined TUs were then categorized into mono-cistronic or poly-cistronic TUs based on the number of associated genes. For TUs starting from internal TSS, the TSS assigned gene was not considered as ‘associated’. TUs lacking associated genes were classified as either *cis*-regulatory or sRNA based on the distance from TSS to start position of downstream gene (< 500 bp).

## Supplementary Information

**Additional file 1: Figure S1.** Validation of the determined transcription start sites using RNA-Seq results. (a) RNA-Seq mapping statistics. (b) PCA analysis of RNA-Seq mapping results. (c) RNA-Seq read density near transcription start sites. M, T, L and S denote for mid exponential phase, transition phase, late exponential phase and stationary phase, respectively. **Figure S2.** Promoter sequence diversity according to the genetic function. The primary and secondary TSSs of COG assigned genes were used for motif discovery. When the TSSs of a certain COG category is less than 20, the category was excluded for motif discovery. If the number of TSSs associated to discovered motif is less than half of the number of the TSSs used for motif discovery, the discovered motif was excluded. **Figure S3.** Identification of sigma factor recognition motifs. (a) The potential binding motif and regulon of SAVERM_741. (b) The potential binding motif and regulon of SAVERM_3117. The potential regulons of each sigma factor are presented below each predicted motif. Genes annotated as ‘hypothetical protein’ were not presented. **Figure S4.** Analysis on SARP-family regulators. (a) Conserved SARP binding heptameric sequence across the Streptomyces. Unique heptameric sequences were used to create the sequence logo. (b) Predicted binding sites of SARP family regulators. (c) Expression change of the identified SARP family regulators and other genes located in the same BGCs. Genes are listed in the order of expression fold change value at stationary phase. Regulators are colored in red. Genes with expression fold change *P*-value > 0.05 (DESeq2) in all time points are represented with dotted lines. M, T, L and S denote for mid exponential phase, transition phase, late exponential phase and stationary phase, respectively. **Figure S5.** Features of TEPs. (a) RNA-Seq read density near TEPs. (b) Nucleotide usage near TEPs. **Figure S6.** Determined TSSs, TEPs and TUs of secondary metabolite biosynthesis gene clusters. The second line of each BGC is the putative product predicted by antiSMASH and the actual or predicted products are additionally written in red if the antiSMASH prediction is inaccurate.

**Additional file 2: Table S1.** List of transcription start sites (TSSs) identified in this study.

**Additional file 3: Table S2.** Transcription levels of genes determined by RNA-Seq.

**Additional file 4: Table S3.** List of transcript 3′-end positions (TEPs) identified in this study.

**Additional file 5: Table S4.** List of transcription units (TUs) identified in this study.

## Data Availability

The datasets supporting the conclusions of this article are available in the NCBI Gene Expression Omnibus (GEO) repository (GSE118597) (https://www.ncbi.nlm.nih.gov/geo/query/acc.cgi?acc=GSE118597).

## Abbreviations

BGC: Biosynthetic gene cluster
NGS: Next-Generation Sequencing
TU: Transcription unit
TSS: Transcription start site
TEP: Transcript 3′-end position
dRNA-Seq: Differential RNA-Seq
ORF: Open reading frame
UTR: Untranslated region
RBS: Ribosome binding site
CDS: Coding sequence
RPF: Ribosome-protected mRNA fragment
COG: Clusters of Orthologous Groups
BTAD: Bacterial transcriptional activator domain
SARP: *Streptomyces* antibiotic regulatory protein
RPKM: Reads per kilobase per million
TAP: RNA 5′ polyphosphatase
