# Supplementary material for: Genome-scale analysis of genetic regulatory elements in Streptomyces avermitilis MA-4680 using transcript boundary information
Source: BMC Genomics. 2022 Jan 21;23:68. doi: 10.1186/s12864-022-08314-0 (PMC8780764; doi:10.1186/s12864-022-08314-0)
Supplement: Supplementary file 1 — Additional file 1: Figure S1. Validation of the determined transcription start sites using RNA-Seq results. (a) RNA-Seq mapping statistics. (b) PCA analysis of RNA-Seq mapping results. (c) RNA-Seq read density near transcription start sites. M, T, L and S denote for mid exponential phase, transition phase, late exponential phase and stationary phase, respectively. Figure S2. Promoter sequence diversity according to the genetic function. The primary and secondary TSSs of COG assigned genes were used for motif discovery. When the TSSs of a certain COG category is less than 20, the category was excluded for motif discovery. If the number of TSSs associated to discovered motif is less than half of the number of the TSSs used for motif discovery, the discovered motif was excluded. Figure S3. Identification of sigma factor recognition motifs. (a) The potential binding motif and regulon of SAVERM_741. (b) The potential binding motif and regulon of SAVERM_3117. The potential regulons of each sigma factor are presented below each predicted motif. Genes annotated as ‘hypothetical protein’ were not presented. Figure S4. Analysis on SARP-family regulators. (a) Conserved SARP binding heptameric sequence across the Streptomyces. Unique heptameric sequences were used to create the sequence logo. (b) Predicted binding sites of SARP family regulators. (c) Expression change of the identified SARP family regulators and other genes located in the same BGCs. Genes are listed in the order of expression fold change value at stationary phase. Regulators are colored in red. Genes with expression fold change P-value > 0.05 (DESeq2) in all time points are represented with dotted lines. M, T, L and S denote for mid exponential phase, transition phase, late exponential phase and stationary phase, respectively. Figure S5. Features of TEPs. (a) RNA-Seq read density near TEPs. (b) Nucleotide usage near TEPs. Figure S6. Determined TSSs, TEPs and TUs of secondary metabolite biosynthesis gene cluster [file 12864_2022_8314_MOESM1_ESM.docx]

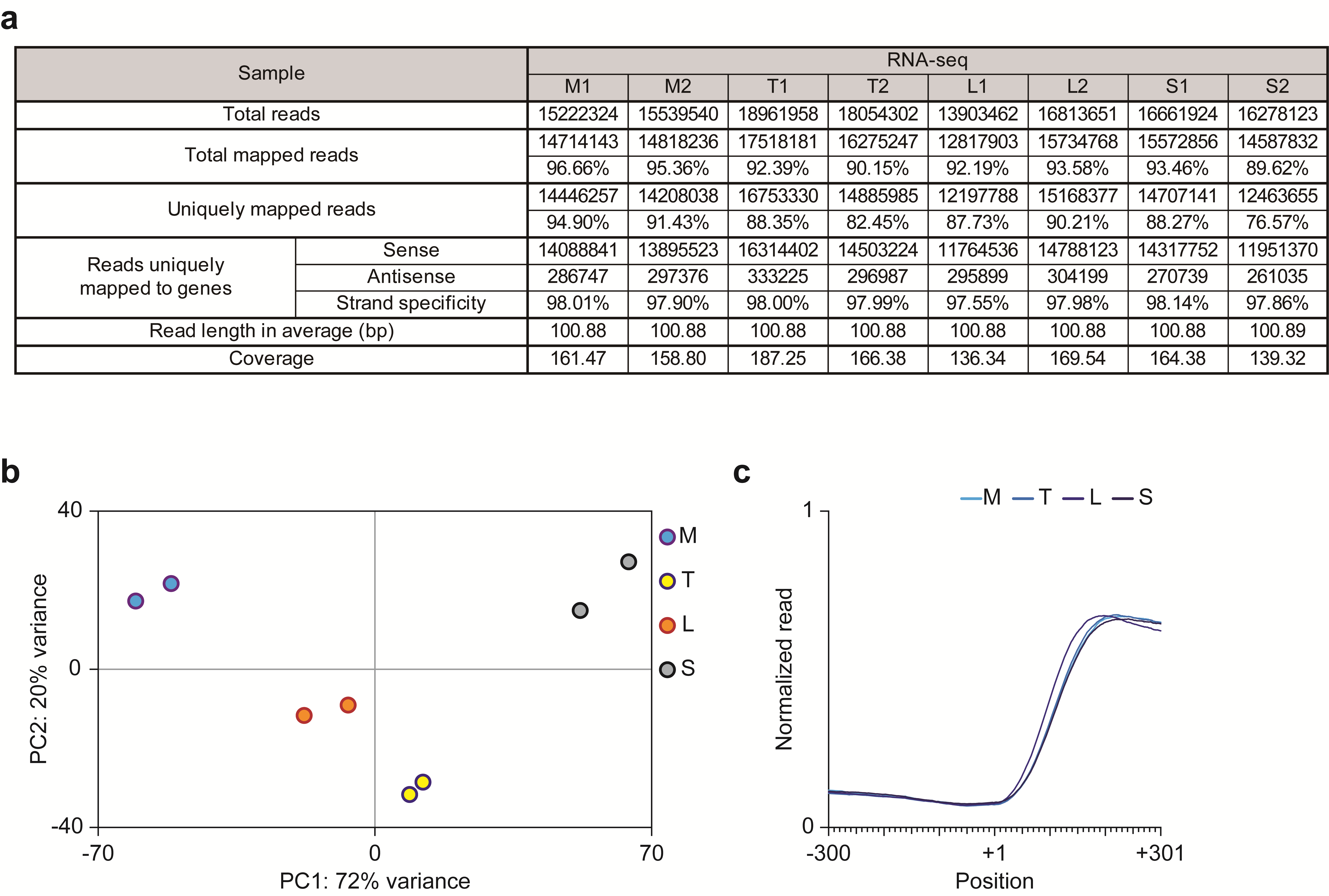


**Figure S1.** Validation of the determined transcription start sites using RNA-Seq results. (a) RNA-Seq mapping statistics. (b) PCA analysis of RNA-Seq mapping results. (c) RNA-Seq read density near transcription start sites. M, T, L and S denote for mid exponential phase, transition phase, late exponential phase and stationary phase, respectively.


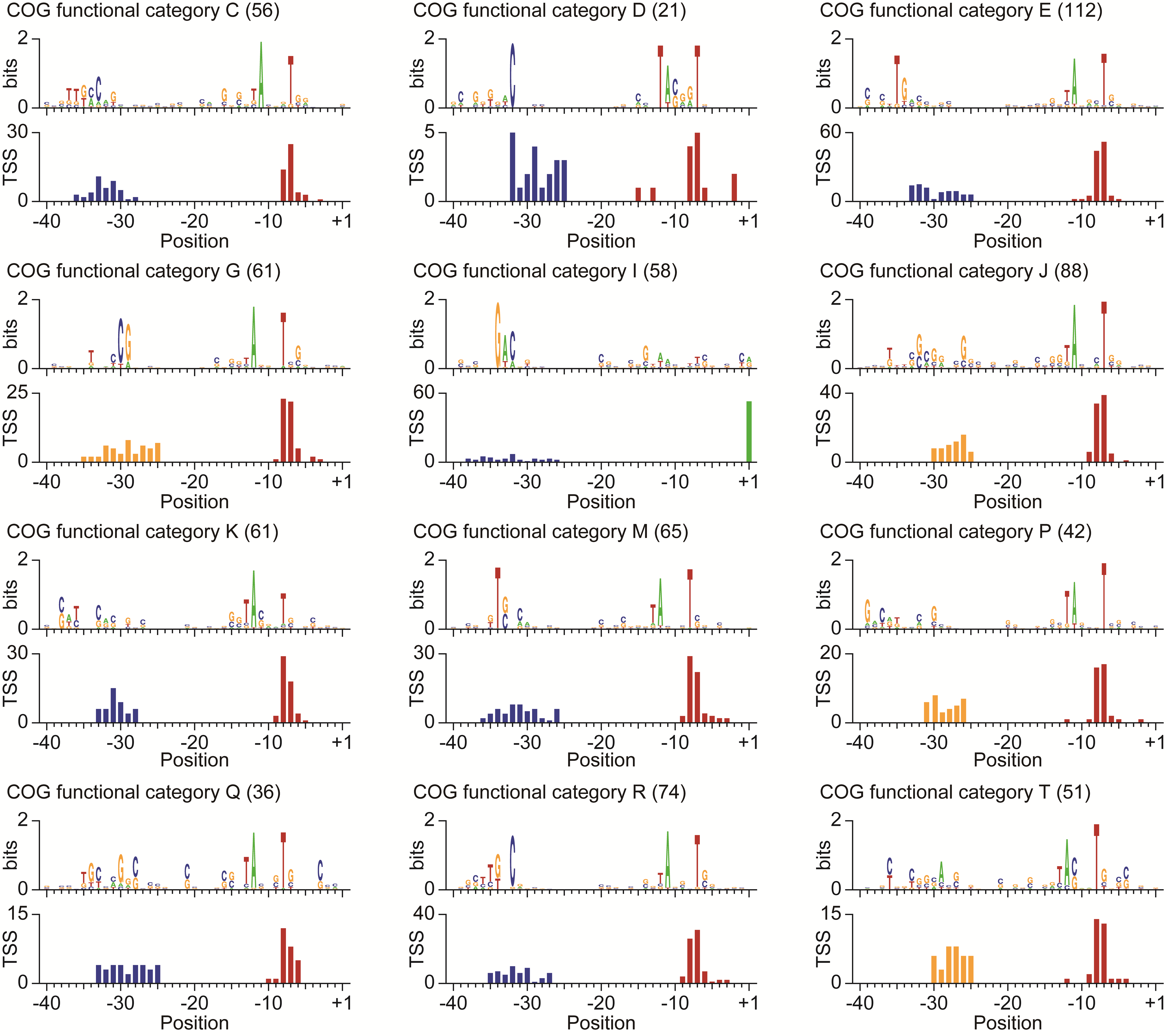


**Figure S2.** Promoter sequence diversity according to the genetic function. The primary and secondary TSSs of COG assigned genes were used for motif discovery. When the TSSs of a certain COG category is less than 20, the category was excluded for motif discovery. If the number of TSSs associated to discovered motif is less than half of the number of the TSSs used for motif discovery, the discovered motif was excluded.


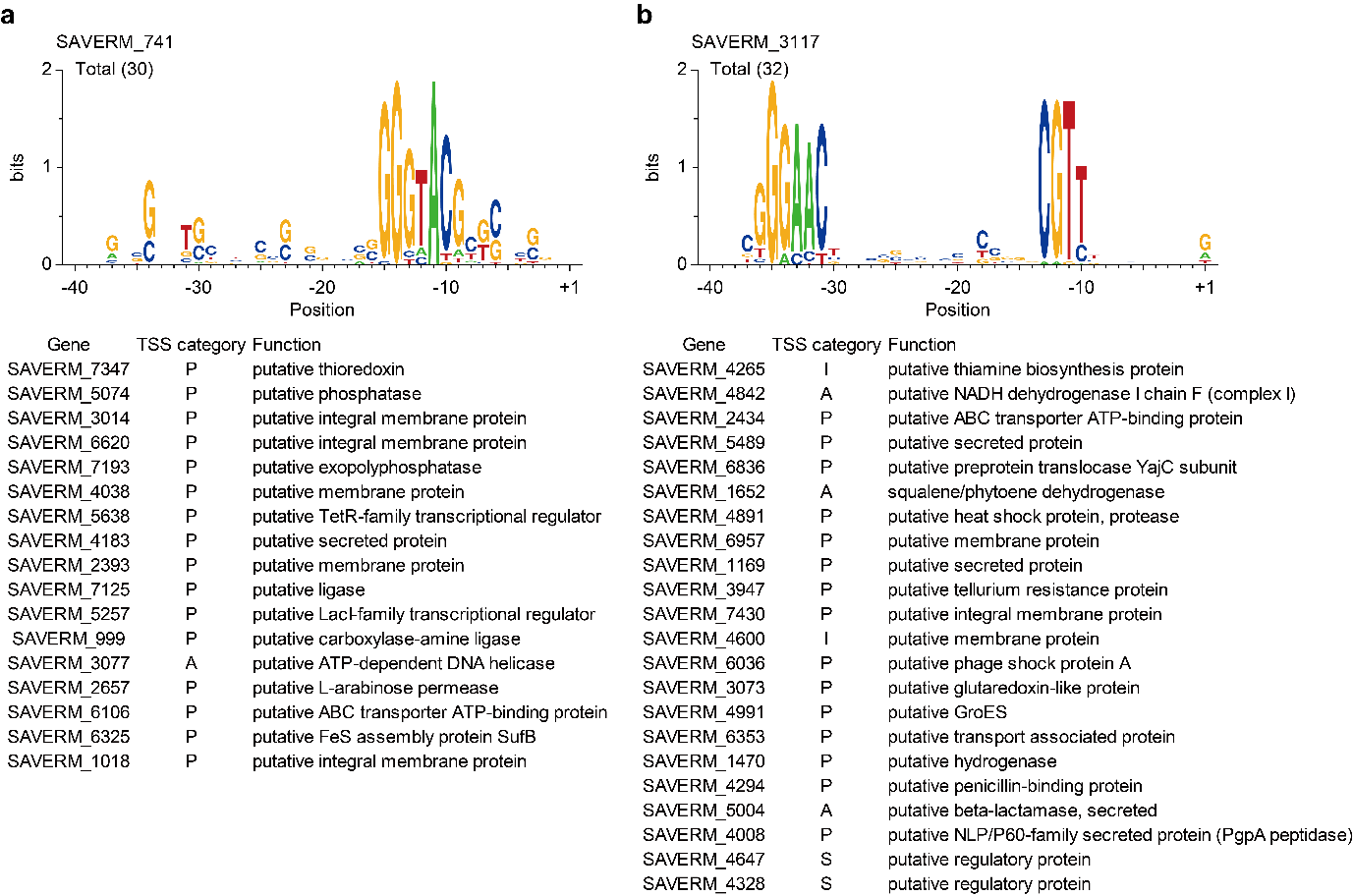


**Figure S3.** Identification of sigma factor recognition motifs. (a) The potential binding motif and regulon of SAVERM_741. (b) The potential binding motif and regulon of SAVERM_3117. The potential regulons of each sigma factor are presented below each predicted motif. Genes annotated as ‘hypothetical protein’ were not presented.





**Figure S4.** Analysis on SARP-family regulators. (a) Conserved SARP binding heptameric sequence across the Streptomyces. Unique heptameric sequences were used to create the sequence logo. (b) Predicted binding sites of SARP family regulators. (c) Expression change of the identified SARP family regulators and other genes located in the same BGCs. Genes are listed in the order of expression fold change value at stationary phase. Regulators are colored in red. Genes with expression fold change *P*-value > 0.05 (DESeq2) in all time points are represented with dotted lines. M, T, L and S denote for mid exponential phase, transition phase, late exponential phase and stationary phase, respectively.


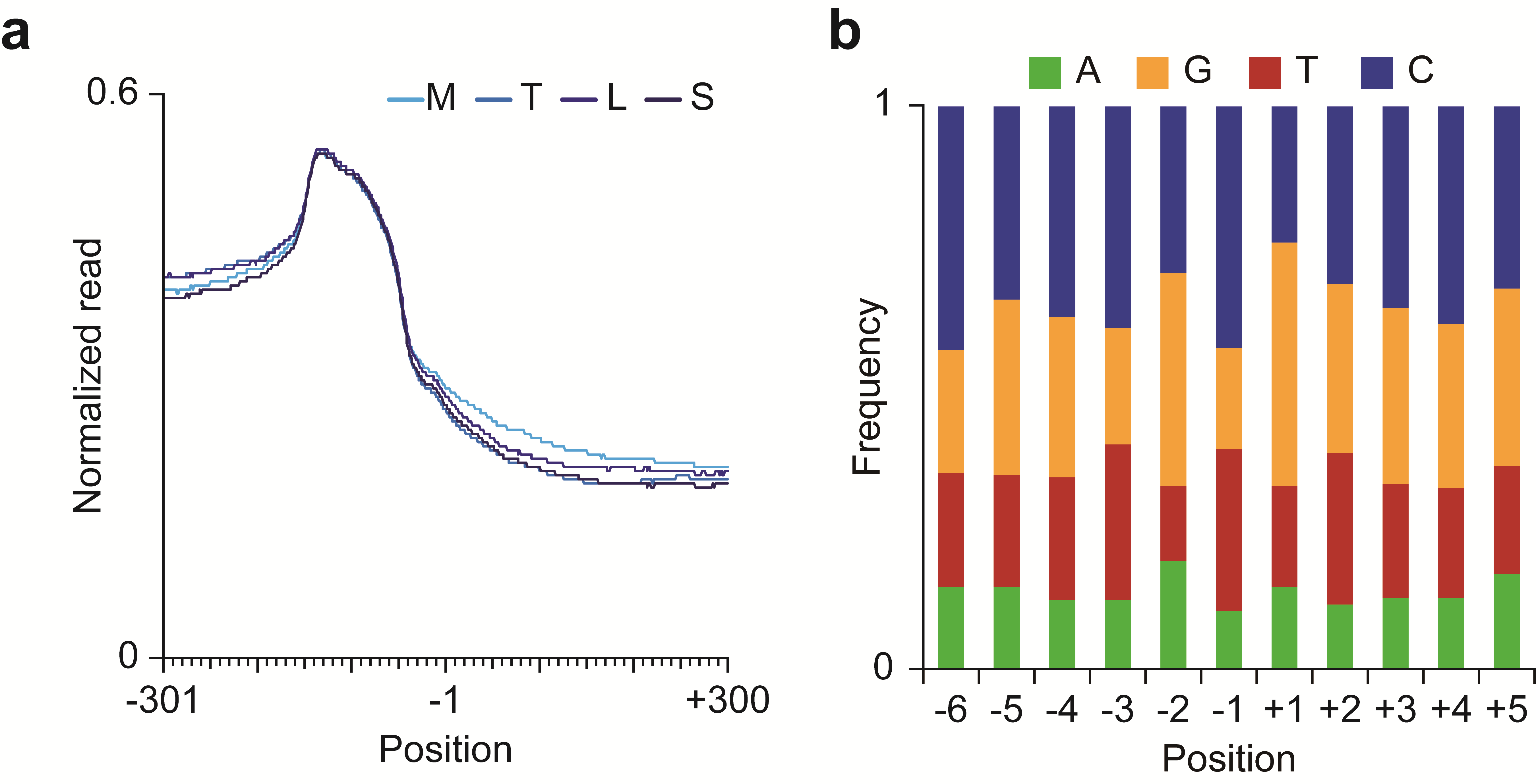


**Figure S5.** Features of TEPs. (a) RNA-Seq read density near TEPs. (b) Nucleotide usage near TEPs.





**Figure S6.** Determined TSSs, TEPs and TUs of secondary metabolite biosynthesis gene clusters. The second line of each BGC is the putative product predicted by antiSMASH and the actual or predicted products are additionally written in red if the antiSMASH prediction is inaccurate.
